# Bridging mechanism and design: modern medicinal chemistry approaches to thymidylate synthase inhibitors

**DOI:** 10.1039/d5ra08381h

**Published:** 2026-01-16

**Authors:** Ahmed A. Al-Karmalawy, Mohamed E. Eissa, Tarek A. Yousef, Arwa Omar Al Khatib, Samia S. Hawas

**Affiliations:** a Department of Pharmaceutical Chemistry, College of Pharmacy, The University of Mashreq Baghdad 10023 Iraq akarmalawy@horus.edu.eg; b College of Science, Chemistry Department, Imam Mohammad Ibn Saud Islamic University (IMSIU) Riyadh 11623 Saudi Arabia tayousef@imamu.edu.sa; c Faculty of Pharmacy, Hourani Center for Applied Scientific Research, Al-Ahliyya Amman University Amman Jordan; d Department of Pharmaceutical Chemistry, Faculty of Pharmacy, Horus University-Egypt New Damietta 34518 Egypt

## Abstract

Thymidylate synthase (TS) is a folate-dependent enzyme essential for DNA synthesis and cell proliferation, making it one of the most enduring and clinically validated targets in anticancer chemotherapy. This review provides a comprehensive overview of TS structure, catalytic mechanism, and inhibition modes, emphasizing its central role in the action of classical antimetabolites such as fluoropyrimidines and antifolates. Despite decades of clinical success, drug resistance, toxicity, and limited tumor selectivity continue to challenge TS-targeted therapy. Recent advances in medicinal chemistry have introduced novel heterocyclic scaffolds, particularly oxadiazoles, pyrimidines, and their hybrid analogs, exhibiting improved TS inhibition, cytotoxic selectivity, and multitarget potential. Structure–activity relationship (SAR) analyses reveal key molecular features governing potency, including halogen substitution, π–π stacking interactions, and bioisosteric modifications. Moreover, innovative strategies such as noncatalytic enzyme destabilizers, hybrid multitarget inhibitors, and biomarker-guided prodrug systems are reshaping the future of TS-directed therapeutics. This review highlights the structural evolution of TS inhibitors from classical to next-generation agents, bridging mechanistic understanding with the design of safer and more effective anticancer drugs.

## Abstract 2

Modern medicinal chemistry approaches to thymidylate synthase inhibitors.

## Graphical abstract

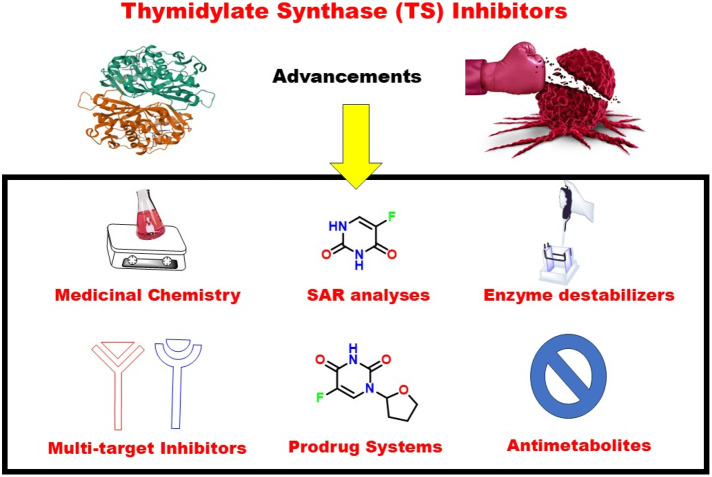

## 1 Introduction

Thymidylate synthase (TS; EC 2.1.1.45) is a key folate-dependent enzyme that catalyzes the reductive methylation of deoxyuridine monophosphate (dUMP) to deoxythymidine monophosphate (dTMP), a critical step in the *de novo* biosynthesis of thymidylate, the sole precursor for thymidine triphosphate (dTTP) required for DNA replication and repair.^1–4^ Because of its indispensable role in maintaining genomic integrity, TS is considered an essential enzyme for cellular proliferation and survival.^5,6^ Aberrant regulation or overexpression of TS has been observed in several human malignancies, correlating with enhanced tumor aggressiveness and poor clinical outcome.^7–10^

Historically, TS was among the earliest molecular targets exploited in cancer chemotherapy.^11–13^ Antimetabolites such as fluoropyrimidines and antifolates were developed to interfere with thymidylate biosynthesis, leading to imbalances in nucleotide pools and subsequent inhibition of DNA synthesis.^14–18^ These agents, which remain central to many chemotherapeutic regimens, highlight the clinical importance of targeting folate-dependent one-carbon metabolism.^19,20^ Yet, prolonged therapy often leads to drug resistance and dose-limiting toxicities, prompting ongoing efforts to refine the molecular understanding of TS structure, its catalytic mechanism, and modes of inhibition to design more selective and durable therapeutic agents.^21–23^

### 1.1 Structure and catalytic mechanism of thymidylate synthase

**Fig. 1:**
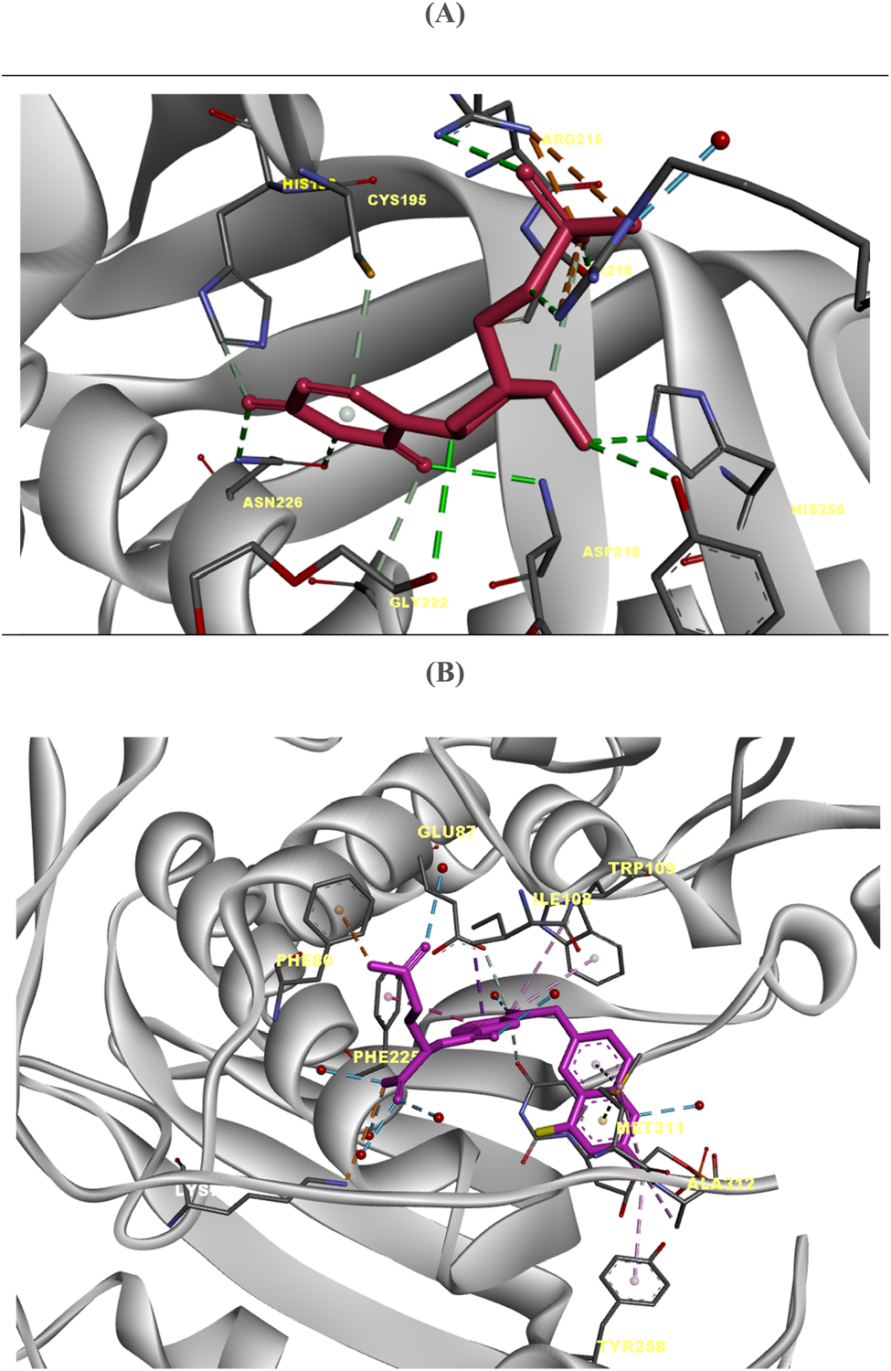
Crystal structures of thymidylate synthase to indicate the catalytic mechanism; (A) crystal structure of His-tag human thymidylate synthase (HT-*h*TS) in complex with dUMP (PDB ID: 6QXH); (B) human thymidylate synthase complexed with dUMP and Raltitrexed, an antifolate drug, is in the closed conformation (PDB ID: 1HVY).

Human TS is a 72 kDa homodimeric enzyme in which each monomer contributes to the formation of the other's active site, underscoring the importance of dimerization for catalytic function.^24^ Each monomer comprises a large, conserved domain that accommodates the folate-binding pocket and a smaller, variable domain associated with substrate recognition.^25,26^ The catalytic cysteine residue (Cys195) located in the flexible loop (residues 184–199) plays a pivotal role in initiating the methyl transfer reaction.^27,28^ Crystallographic analyses reveal that the TS active site undergoes conformational rearrangements between active and inactive states.^29,30^ In the active conformation, Cys195 is positioned within the catalytic pocket, where it forms a covalent bond with the C6 atom of the dUMP substrate.^31,32^ The uracil moiety of dUMP interacts *via* hydrogen bonds with Asn226 and Asp218, while the phosphate group is stabilized by a network of arginine residues from both monomers^33,34^ (Fig. 1A). The binding of the cofactor 5,10-methylene-5,6,7,8-tetrahydrofolate (mTHF) follows, enabling the sequential transfer of a methyl group to yield dTMP and dihydrofolate (DHF).^35^ TS also exhibits autoregulatory capacity through direct interaction with its own mRNA, a feature that contributes to translational control and has implications for drug-induced feedback regulation.^36^ The enzyme can adopt inactive conformations in which the catalytic loop is rotated outward, preventing Cys195 from participating in catalysis.^37^ Structural studies further demonstrate that inhibitors such as raltitrexed and pemetrexed bind at the folate-binding site above dUMP, engaging residues like Glu87, Ile108, Trp109, and Phe225.^33^ This intricate network of active-site interactions provides a structural basis for rational drug design targeting TS (Fig. 1B).

### 1.2 Mechanism of enzyme inhibition

**Fig. 2:**
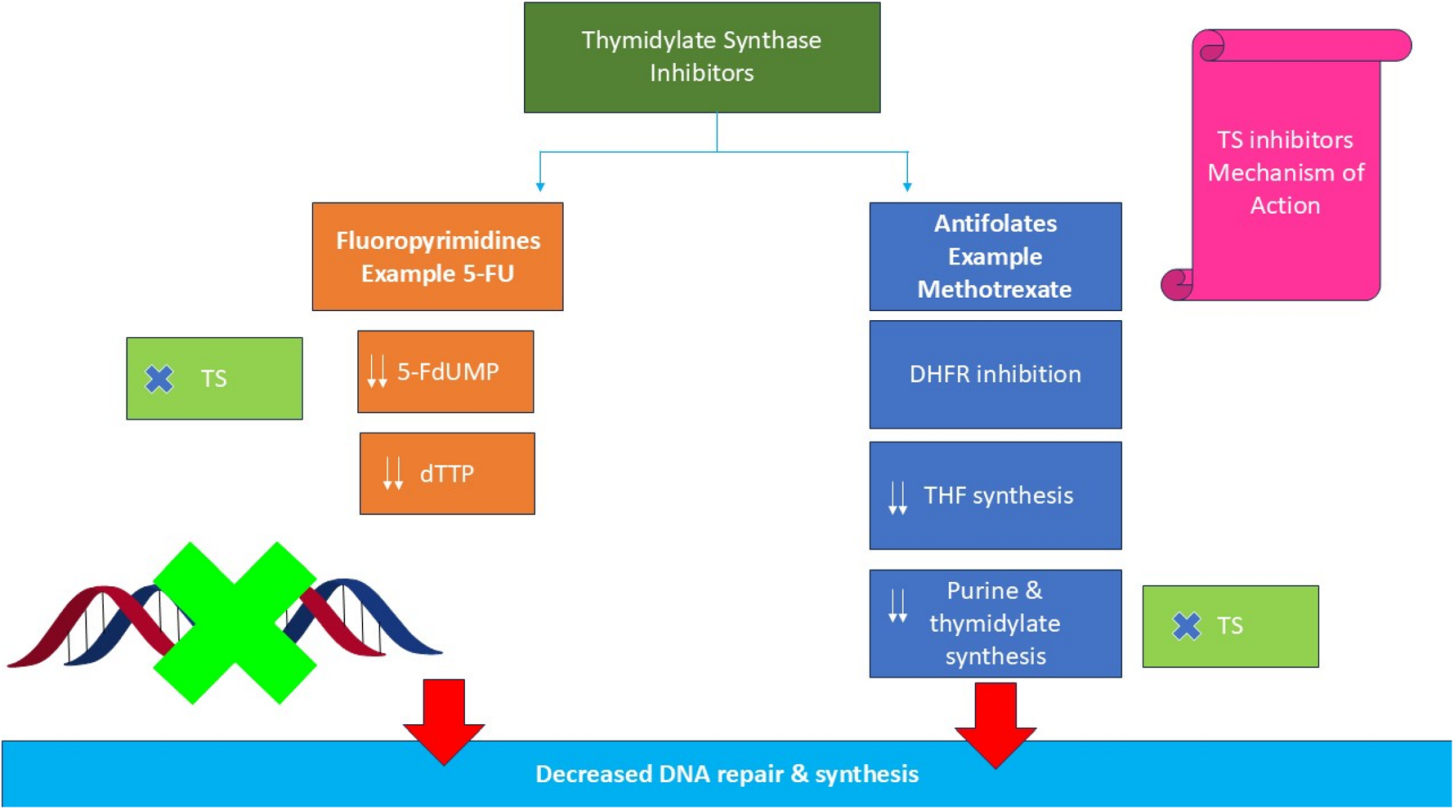
Thymidylate synthase mechanism of action.

The inhibition of TS disrupts the *de novo* thymidylate synthesis pathway, leading to depletion of dTMP and accumulation of dUMP and dUTP.^38–40^ These imbalances result in uracil misincorporation into DNA, triggering futile cycles of base excision repair, DNA strand breaks, and ultimately, cell death.^41–44^ Two major classes of TS inhibitors have been developed: (1) fluoropyrimidines, which mimic the natural substrate dUMP and act as mechanism-based (“suicide”) inhibitors,^45–47^ and (2) antifolates, which compete with the folate cofactor mTHF.^48,49^ Fluoropyrimidines such as 5-fluorouracil (5-FU) and its prodrugs (capecitabine, tegafur) are metabolically converted into 5-fluoro-deoxyuridine monophosphate (FdUMP), which forms a stable ternary complex with TS and mTHF, irreversibly blocking catalysis.^50–52^ In contrast, antifolates such as methotrexate, raltitrexed, pralatrexate, and pemetrexed are structural analogs of folate that bind competitively to the folate-binding site.^53–56^ Many of these agents undergo intracellular polyglutamation by folylpolyglutamate synthetase (FPGS), enhancing their affinity for TS and increasing cellular retention.^57,58^ Through these interactions, antifolates effectively suppress thymidylate synthesis and disrupt other folate-dependent metabolic processes.^59^ However, TS overexpression, enhanced folate transport, and alterations in polyglutamation capacity often contribute to drug resistance, emphasizing the complexity of targeting this enzyme in cancer therapy.^60,61^Fig. 2 illustrates the thymidylate synthase mechanism of action.

### 1.3 Thymidylate synthase-targeting anticancer drugs

TS-targeting agents remain fundamental in the clinical management of a wide spectrum of solid tumors.^62–64^ Fluoropyrimidines, first introduced in the 1950s, revolutionized chemotherapy.^11^ 5-FU, the archetypal member of this class, undergoes intracellular activation to FdUMP, FUTP, and FdUTP. FdUMP inhibits TS, whereas FUTP and FdUTP interfere with RNA and DNA synthesis, respectively.^65–67^ 5-FU is widely used in colorectal, gastric, breast, pancreatic, and head and neck cancers, either as monotherapy or in combination regimens such as FOLFOX and FOLFIRI.^68–72^ Its oral prodrug, capecitabine, was developed to enhance selectivity and convenience, being enzymatically converted to 5-FU preferentially in tumor tissues.^73–76^ Tegafur, another prodrug often combined with modulators like uracil or gimeracil, undergoes hepatic activation to 5-FU and is used in gastrointestinal malignancies.^77–80^

**Fig. 3:**
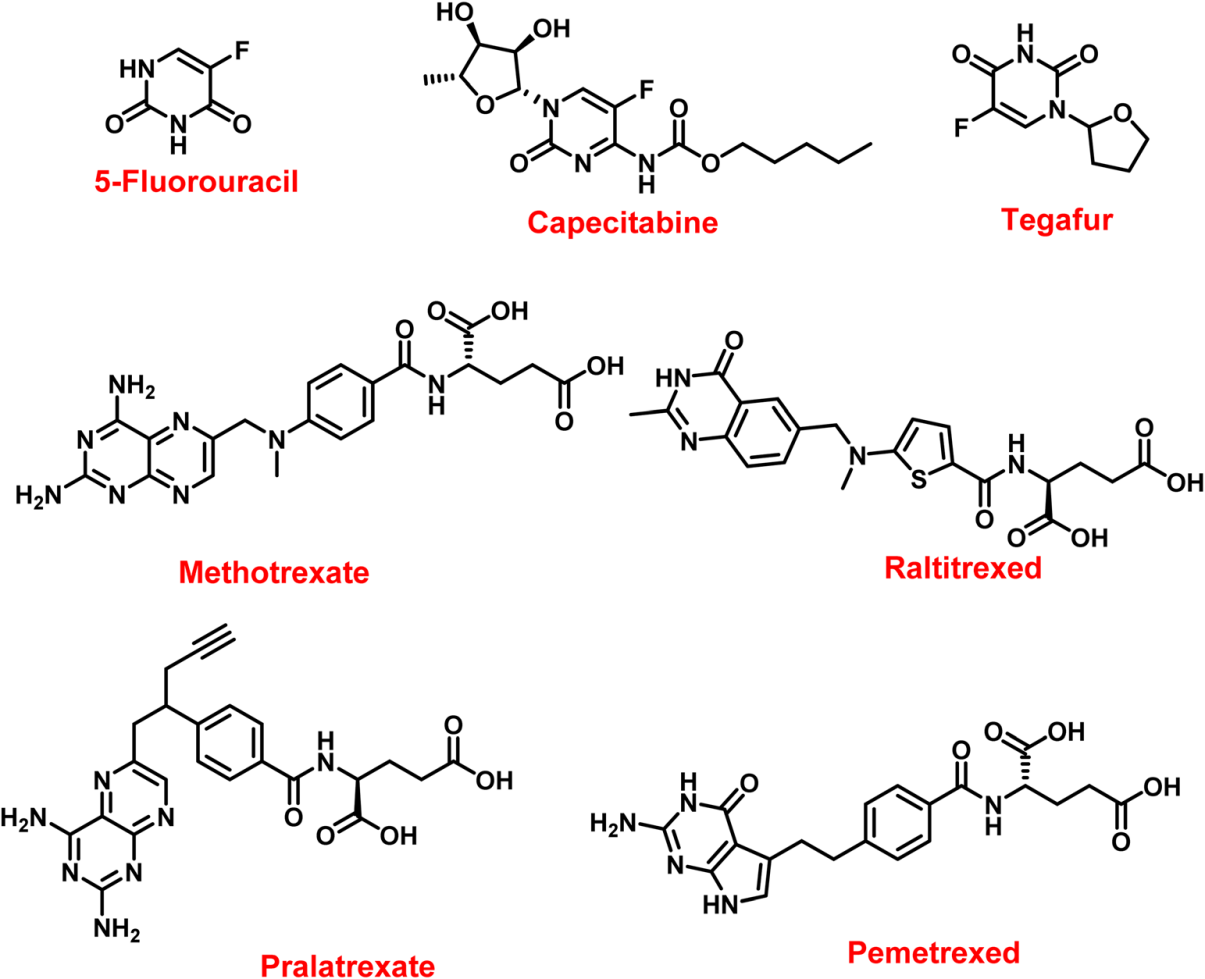
Fluoropyrimidines and antifolates structures.

Among antifolates, methotrexate was the pioneering folate antagonist, initially developed for leukemia and later extended to various cancers and autoimmune diseases.^81–84^ It inhibits dihydrofolate reductase (DHFR), indirectly suppressing TS activity by depleting tetrahydrofolate pools.^85,86^ Raltitrexed, a quinazoline-based inhibitor, directly targets TS with high selectivity and has been approved for colorectal cancer and mesothelioma.^87–92^ Pralatrexate, designed to exhibit enhanced affinity for the reduced folate carrier (RFC) and efficient polyglutamation, has shown superior uptake and retention compared with methotrexate and is approved for relapsed peripheral T-cell lymphoma.^89–94^ Pemetrexed represents a new generation of multitarget antifolates that inhibit TS, DHFR, and glycinamide ribonucleotide formyltransferase (GARFT).^91,92,95,96^ It is clinically approved for non-small cell lung cancer and malignant pleural mesothelioma.^97^ Polyglutamated pemetrexed forms exhibit markedly increased potency, and their accumulation in the acidic tumor microenvironment *via* proton-coupled folate transporter (PCFT) enhances selective cytotoxicity.^98,99^ Despite their success, these drugs are limited by intrinsic or acquired resistance driven by TS overexpression, altered folate transport, and changes in one-carbon metabolism.^100^Fig. 3 and Table 1 show classes of thymidylate synthase inhibitors.

**Table 1:** Classes of thymidylate synthase inhibitors

Class	Drug	Mechanism of action	Clinical use/Approved indications	Key features	References
Fluoropyrimidines	5-Fluorouracil (5-FU)	Converted intracellularly to FdUMP, FUTP, and FdUTP; FdUMP irreversibly inhibits TS, while FUTP and FdUTP disrupt RNA and DNA synthesis	Colorectal, gastric, breast, pancreatic, and head & neck cancers; used in FOLFOX, FOLFIRI regimens	Prototype TS inhibitor; cornerstone of fluoropyrimidine therapy	68–72
	Capecitabine	An oral prodrug is enzymatically converted to 5-FU within tumor tissues	Colorectal and breast cancers	Enhanced tumor selectivity and oral convenience	73–76
	Tegafur (±uracil or gimeracil)	Hepatically metabolized to 5-FU; modulators (uracil/gimeracil) inhibit catabolism, prolonging 5-FU exposure	Gastrointestinal malignancies	Improved bioavailability; reduced systemic toxicity	77–80
Antifolates	Methotrexate (MTX)	Inhibits dihydrofolate reductase (DHFR) → indirect TS inhibition *via* tetrahydrofolate depletion	Leukemia, breast, head & neck cancers, osteosarcoma; autoimmune diseases	First folate antagonist; affects one-carbon metabolism	81–84
	Raltitrexed	Direct TS inhibitor (quinazoline-based)	Colorectal cancer, mesothelioma	High selectivity for TS; folate-based structure	87–92, 101 and 102
	Pralatrexate	Inhibits DHFR and TS; high affinity for reduced folate carrier (RFC) and enhanced polyglutamation	Relapsed/refractory peripheral T-cell lymphoma	Superior uptake and retention *vs.* MTX	89–94
	Pemetrexed	Multi-target antifolate: Inhibits TS, DHFR, and GARFT	Non-small cell lung cancer; malignant pleural mesothelioma	Polyglutamated forms show increased potency; selective tumor uptake *via* PCFT	91, 92, 95 and 96

### 1.4 Concluding perspective

Over six decades of research have positioned TS as a cornerstone of antimetabolite-based chemotherapy.^1,63^ The enzyme's structural features, multifaceted regulation, and integration into folate metabolism provide multiple opportunities for therapeutic intervention.^2,62,103,104^ However, the adaptability of cancer cells through TS overexpression and compensatory pathway activation continues to limit the long-term success of current inhibitors.^105^

Future strategies integrating structural biology, systems pharmacology, and medicinal chemistry are essential for developing next-generation TS-targeted agents capable of overcoming resistance and achieving durable clinical responses.

## 2 Reported thymidylate synthase inhibitors and their structure–activity relationships study

Recent years have witnessed significant progress in the design of TS inhibitors incorporating heterocyclic scaffolds such as oxadiazoles and pyrimidines. These frameworks offer diverse hydrogen-bonding, π–π stacking, and electronic properties that enhance enzyme binding and cytotoxic activity. The following section summarizes key structure–activity relationship (SAR) trends and the most potent compounds reported in recent studies.

### 2.1 Oxadiazole-based thymidylate synthase inhibitors

The 1,3,4-oxadiazole nucleus has emerged as a privileged scaffold in TS inhibitor design owing to its planarity, bioisosteric resemblance to amide and ester functionalities, and capacity to engage in hydrogen bonding and π–π stacking interactions. Several oxadiazole-containing hybrids have demonstrated remarkable TS inhibition and cytotoxic activity against diverse cancer cell lines.^106^ The key SAR findings for different oxadiazole-based TS inhibitor classes are discussed below.

**Fig. 4:**
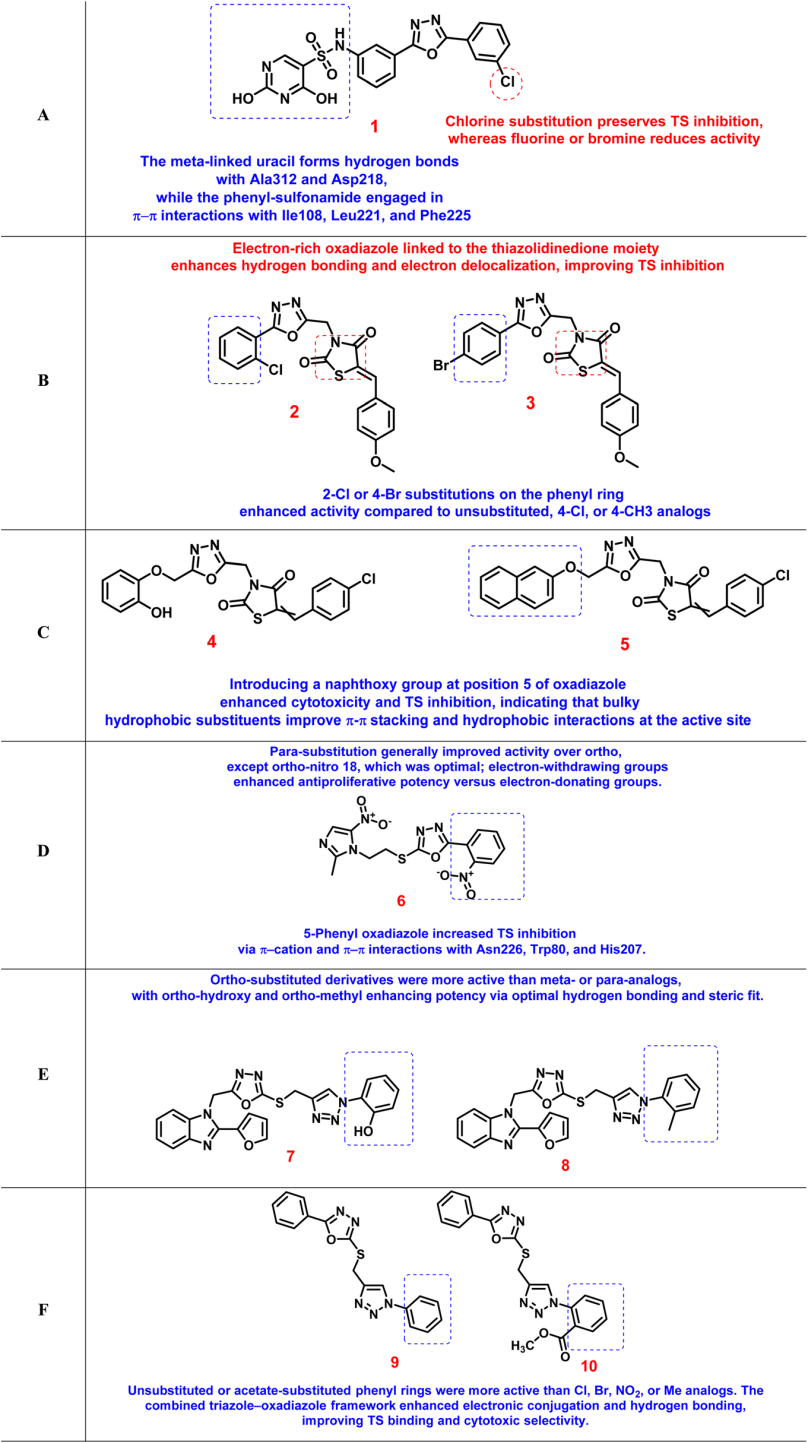
Structure–activity relationships of oxadiazole-based thymidylate synthase inhibitors.

A new series of *N*-(3-(5-phenyl-1,3,4-oxadiazol-2-yl)phenyl)-2,4-dihydroxypyrimidine-5-sulfonamide derivatives was developed as dual TS and antiangiogenic agents.^107^ Among these, compound 1 emerged as the most potent TS inhibitor (IC_50_ = 0.11 µM), exhibiting pronounced selectivity toward non-small cell lung cancer (NSCLC) cell lines. SAR analysis revealed that fluorine or bromine substitution at the terminal phenyl ring (5-position of the oxadiazole) led to a reduction in antiproliferative activity, likely due to increased hydrophobicity or steric interference that hindered optimal binding. In contrast, chlorine substitution was well tolerated and retained significant TS inhibition (Fig. 4A). Molecular docking studies showed that the meta-linked uracil fragment of compound 1 established three strong hydrogen bonds with Ala312 and Asp218, while the phenyl-sulfonamide moiety engaged in π–π stacking with Ile108, Leu221, and Phe225. These interactions rationalized the superior binding affinity of 1 relative to pemetrexed (PTX) and underscored the critical influence of halogen type and position on TS affinity (SI, Fig. S1A).

In another study, a library of thiazolidinedione–1,3,4-oxadiazole hybrids was synthesized and evaluated as TS inhibitors.^108^ Compounds 2 and 3 exhibited 4.5- and 4.4-fold higher activity than 5-FU against MCF-7 cells and 3.1- and 2.5-fold higher cytotoxicity against HCT-116 cells, with TS inhibitory IC_50_ values of 1.67 and 2.21 µM, respectively. SAR observations suggested that conjugating the electron-rich oxadiazole with a thiazolidinedione moiety at the C2 position enhanced hydrogen-bonding interactions and electronic delocalization, thereby strengthening enzyme inhibition. Furthermore, substitution at the 5-position of the oxadiazole ring affected lipophilic–hydrophilic balance, influencing both cell permeability and TS affinity. The presence of 2-Cl or 4-Br substituents on the phenyl ring increased activity compared to unsubstituted, 4-Cl, or 4-CH_3_ derivatives (Fig. 4B). In compound 2, the oxygen of the 4-methoxy substituent participates in hydrogen bonding with Cys195 and Tyr135, while the oxadiazole ring oxygen interacts with Asn226. In compound 3, the methoxy oxygen instead forms a hydrogen bond with Tyr230. Additionally, the thiazolidinedione carbonyl at the 4-position engages in hydrogen bonding with Asn226, and the oxadiazole ring nitrogen at the 4-position interacts with Leu221 (SI, Fig. S1B).

A related scaffold, thiazolidine-2,4-dione-oxadiazole hybrids with dual PPAR-γ agonistic and TS inhibitory properties, was subsequently designed.^109^ Compounds 4 and 5 demonstrated TS inhibitory IC_50_ values of 5.1 and 3.2 µM, respectively. Incorporation of a naphthoxy substituent at the 5-position of the oxadiazole markedly enhanced both cytotoxic and TS inhibitory potency. This observation indicated that bulky hydrophobic substituents can improve π–π stacking and hydrophobic interactions within the TS active site (Fig. 4C). Thus, both electronic and steric modifications of the phenoxy ring were found to govern the delicate balance between receptor activation and TS inhibition. Compound 4 displayed a π–π cation interaction with Ala111, Met311, Ala312, and Arg50, along with a hydrogen-bonding interaction with Cys195. In contrast, compound 5 was stabilized within the TS binding pocket through interaction with Tyr258 and formed π–π cation interactions with Ala312, Arg215, Cys195, and Ala111, in addition to hydrogen bonds with His256, Tyr258, and Cys195 (SI, Fig. S1C).

A distinct set of 1,3,4-oxadiazole thioether derivatives was reported as dual antitumor and antibacterial TS inhibitors.^110^ Compound 6, bearing an *ortho*-nitro substituent, was the most potent with IC_50_ values of 0.62 µM (human TS) and 0.47 µM (*E. coli* TS), alongside notable anticancer activity (IC_50_ = 0.7 µM). SAR analysis revealed that *para*-substituted derivatives generally exhibited higher activity than *ortho*-substituted analogs, except for one compound, whose *ortho*-nitro group provided optimal electronic complementarity with catalytic residues. Electron-withdrawing substituents consistently enhanced antiproliferative potency compared with electron-donating groups. Moreover, replacement of the 5-position of the oxadiazole ring with a phenyl substituent enhanced TS inhibition (Fig. 4D). Docking results demonstrated strong π–cation and π–π interactions with Asn226, Trp80, and His207, supporting the experimental findings (SI, Fig. S1D).

Another promising scaffold involved benzimidazole-1,3,4-oxadiazole hybrids.^111^ Compounds 7 and 8 showed potent TS inhibition (IC_50_ ≈ 1 µM) and selective cytotoxicity toward A549, SKOV3, and MDA-MB-231 cell lines. SAR analysis revealed that *ortho*-substituted derivatives were generally more active than *meta*- or *para*-substituted analogs. Specifically, *ortho*-hydroxy and *ortho*-methyl groups improved potency, likely by promoting favorable hydrogen bonding and achieving a sterically optimized orientation within the enzyme active site (Fig. 4E). Compound 7 formed hydrogen bonding with Asp218, Cys195, and Asn226, and only Cys195 for compound 8 (SI, Fig. S1E).

Finally, a series of 1,2,3-triazole-1,3,4-oxadiazole hybrids was investigated for TS inhibition and anticancer potential.^112^ Compounds 9 and 10 exhibited four- and five-fold higher TS inhibition compared with 5-FU in MCF-7 cells, with IC_50_ values of 2.52 and 4.38 µM, respectively. The synergistic combination of triazole and oxadiazole rings was found to enhance electronic conjugation and hydrogen-bonding ability, thereby strengthening interactions within the TS binding pocket. Unsubstituted or acetate-substituted phenyl rings conferred superior activity compared with those bearing Cl, Br, NO_2_, or CH_3_ groups (Fig. 4F). This study confirmed that the hybridization of heterocyclic moieties significantly improves enzyme affinity and cytotoxic selectivity, underscoring the pharmacophoric potential of oxadiazole scaffolds in TS inhibitor design. The nitrogen atom of the 1,2,3-triazole ring in compounds 9 and 10 forms hydrogen-bonding interactions with the Asn226 residue. In compound 10, two additional π–π interactions are observed: one between the 1,2,3-oxadiazole ring and HIE196, and another involving the 1,2,3-triazole ring and the Phe225 residue (SI, Fig. S1F). Table 2 shows a summary of oxadiazole-based thymidylate synthase inhibitors.

**Table 2:** Summary of oxadiazole-based thymidylate synthase inhibitors

Compound(s)	Scaffold/type	IC_50_ (TS or cytotoxicity)	SAR highlights	References
1	*N*-(3-(5-Phenyl-1,3,4-oxadiazol-2-yl)phenyl)-2,4-dihydroxypyrimidine-5-sulfonamides	0.11 µM	Cl tolerated; F/Br decreased activity; uracil formed H-bonds with Ala312, Asp218	107
2, 3	Thiazolidinedione-1,3,4-oxadiazole hybrids	1.67–2.21 µM (TS)	C2-thiazolidinedione improved H-bonding; 2-Cl or 4-Br > 4-Cl/CH_3_	108
4, 5	Thiazolidine-2,4-dione-oxadiazole hybrids	3.2–5.1 µM	Naphthoxy at the 5-position increased potency; bulky hydrophobic substituents enhanced π–π stacking	109
6	1,3,4-Oxadiazole thioethers	0.62 µM (hTS), 0.47 µM (*E. coli* TS)	Electron-withdrawing > donating groups; *para*-substitution favored; π–π and π-cation interactions critical	110
7, 8	Benzimidazole-1,3,4-oxadiazole hybrids	∼1 µM	*Ortho*-OH and *ortho*-Me enhanced potency; better orientation and H-bonding	111
9, 10	1,2,3-Triazole-1,3,4-oxadiazole hybrids	2.52–4.38 µM	Triazole-oxadiazole synergy improved binding; unsubstituted/acetate phenyl > halogenated analogs	112

### 2.2 Pyrimidine-based thymidylate synthase inhibitors

Pyrimidine scaffolds represent one of the most significant structural cores in the design of TS inhibitors, owing to their close resemblance to natural pyrimidine substrates and cofactors.^113^ Numerous pyrimidine-derived molecules have been developed to enhance TS inhibition and exhibit strong antiproliferative properties across various cancer cell lines. The following section summarizes the main SAR trends observed for distinct pyrimidine-based TS inhibitor classes.

**Fig. 5:**
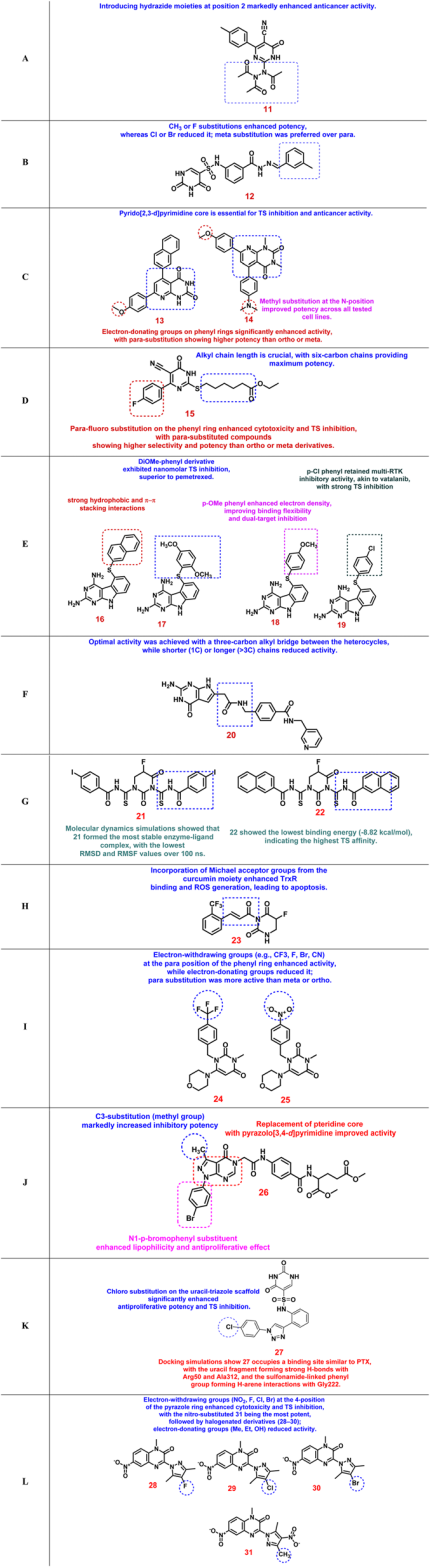
Structure–activity relationships of pyrimidine-based thymidylate synthase inhibitors.

A novel class of 6-aryl-5-cyano-pyrimidine derivatives demonstrated potent antiproliferative and TS inhibitory activities, with IC_50_ values ranging from 3.89 to 15.74 nM.^114^ The most active compound, 11, significantly increased the Bax/Bcl-2 ratio (44-fold) and activated caspase-3, confirming its pro-apoptotic mechanism. Structural optimization revealed that the introduction of hydrazide moieties at position 2 of the pyrimidine ring markedly improved anticancer potency, highlighting the role of hydrogen-bond donor groups in enzyme recognition and apoptotic signaling (Fig. 5A). It established four hydrogen bonds overall: one between the acetyl oxygen and Asn226; two involving the pyrimidinone oxygen, which was anchored by Asp218 and Gly222; and a final hydrogen bond formed between the cyano group at the 5-position of the pyrimidine ring and Ser216 (SI, Fig. S2A).

In another study, a series of (*E*)-*N*-(2-benzylhydrazine-1-carbonyl)phenyl-2,4-deoxy-1,2,3,4-tetrahydropyrimidine-5-sulfonamide derivatives was developed to optimize both TS inhibition and antiproliferative activity.^115^ The hit compound 12 (IC_50_ = 17.21 nM) exhibited superior efficacy to pemetrexed (PTX) *in vitro* and *in vivo*, effectively suppressing tumor growth and angiogenesis in NSCLC models. Structure–activity data revealed that aromatic ring substitution with CH_3_ or F significantly enhanced potency, while Cl or Br substitutions reduced activity. Moreover, *meta*-substitution was found to be more favorable than *para*-substitution, and compound 12 (m-CH_3_) displayed the highest antiproliferative activity, outperforming PTX (Fig. 5B). Ser216 formed two strong hydrogen bonds, one with the carbonyl group and the other with the amino group of the uracil moiety. In addition, the phenyl ring of the benzoyl hydrazone showed an H-arene interaction with Gly222, while the second terminal phenyl ring also engaged in an H-arene interaction with Leu221. At the same time, key stabilizing contacts were observed between the acyl hydrazine carbonyl and His196, as well as between the acyl hydrazine's active hydrogen and Trp109 (SI, Fig. S2B).

A further series of pyrido[2,3-*d*]pyrimidine derivatives was synthesized and evaluated against HCT-116, MCF-7, HepG2, and PC-3 cancer cell lines.^116^ Compounds 13 and 14 showed the most potent anticancer and TS inhibitory activities, surpassing raltitrexed. Their IC_50_ values ranged from 1.48 to 5.18 µM across tested cell lines, with TS inhibitory IC_50_ values of 20.47 and 13.48 nM, respectively. Docking studies confirmed strong binding affinities (−10.6 and −9.5 kcal mol^−1^) compared with raltitrexed (−9.4 kcal mol^−1^). The SAR analysis indicated that the pyrido[2,3-*d*]pyrimidine core was critical for TS inhibition, while electron-donating groups on phenyl rings substantially enhanced activity. Methyl substitution at the *N*-position further improved potency across all tested cell lines, and *para*-substitution on the aryl ring yielded superior IC_50_ values relative to *ortho* or *meta* substitution (Fig. 5C). Compound 13 formed a hydrogen bond with Tyr135 and showed extensive hydrophobic interactions with Asn226, Ser216, Phe80, Ile108, Trp109, Tyr135, Leu192, Cys195, Leu221, Phe225, Tyr258, Met311, and Ala312. Similarly, compound 14 formed a hydrogen bond with Asn226 and engaged in hydrophobic interactions with Phe80, Ile108, Trp109, Tyr135, Leu192, Cys195, Leu221, Phe225, Tyr258, Met311, and Ala312 (SI, Fig. S2C).

A new set of cyanothiouracil-based 2-thiopyrimidine derivatives was also reported as effective TS inhibitors.^117^ The lead compound 15 demonstrated strong anticancer potency comparable to 5-FU with significantly lower cytotoxicity toward normal McCoy cells (selectivity index = 115.86 µM). It exhibited IC_50_ values of 10.11 µM (MCF-7) and 22.49 µM (A549). SAR analysis revealed that a six-carbon alkyl chain conferred optimal activity, while *para*-fluoro substitution on the phenyl ring enhanced both cytotoxicity and TS inhibition. *Para*-substituted derivatives were consistently more potent and selective than *ortho* or *meta*-substituted analogs, suggesting favorable orientation and electronic complementarity within the active site (Fig. 5D). It formed hydrogen bonds with Asn226 and Asp218, along with hydrophobic contacts involving Phe225, Val223, Met311, Ala312, Val313, Trp109, Ile108, Cys195, Leu192, and Tyr258 (SI, Fig. S2D).

A distinct scaffold, pyrimido[4,5-*b*]indole-2,4-diamine derivatives, was developed as dual inhibitors of receptor tyrosine kinases (RTKs) and TS.^118^ Substitution on the 5-thioaryl portion of these compounds modulated both targets simultaneously. Compounds 16–19 displayed strong dual inhibitory activity, with compound 17 showing equipotency to semaxanib (in VEGFR-2 assays) and superior TS inhibition (IC_50_ = 1.1 nM) compared with pemetrexed. SAR findings revealed that 2′-naphthyl substitutions provided strong hydrophobic and π–π stacking interactions, while the 2′,5′-dimethoxyphenyl derivative exhibited nanomolar TS inhibition. The 4′-methoxyphenyl substituent increased electron density and flexibility, enhancing dual-target binding, and 4′-chloro substitution maintained RTK inhibition similar to vatalanib while preserving potent TS inhibition (Fig. 5E).

A novel series of 2-amino-4-oxo-6-substituted pyrrolo[2,3-*d*]pyrimidines was synthesized as multitargeted antifolates capable of inhibiting both TS and *de novo* purine biosynthetic enzymes.^119^ Compound 20 emerged as the most active analog, showing submicromolar antiproliferative activity against KB, A549, and HepG2 cells. Mechanistic assays demonstrated that its cytotoxicity was partially reversed by thymidine or adenosine alone, but completely reversed by their combination, confirming dual inhibition of TS, GARFTase, and AICARFTase. The SAR findings emphasized that optimal activity required an alkyl bridge of three carbons between the heterocycles, whereas shorter or longer chains reduced potency. Compound 20 maintained balanced activity across multiple cell lines, making it a promising multitarget antifolate lead with greater than 80% inhibition of colony formation at the highest concentration (Fig. 5F). Docking studies supported these results by showing high affinity for both TS and purine biosynthesis enzymes. The 2-NH_2_ group of compound 20 forms a hydrogen bond with the backbone of Ala312, while the N3 nitrogen engages in a hydrogen bond with the side chain of Asp218. The N7 nitrogen also participates in hydrogen bonding through its interaction with the side chain of Asn112. In addition, the side-chain amide carbonyl oxygen of compound 20 forms a hydrogen bond with the indole nitrogen of Trp109 (SI, Fig. S2E)

A comprehensive study of pyrimidinedione derivatives inspired by 5-FU led to the design of 81 new molecules containing thiourea and benzoyl groups.^120^ Computational studies identified five derivatives with stronger binding affinities than 5-FU. Conjugation of thiourea and benzoyl functionalities enhanced hydrogen bonding and π–π stacking within the TS binding pocket. Compound 21 demonstrated the most stable enzyme–ligand complex based on molecular dynamics (RMSD/RMSF analyses), whereas compound 22 exhibited the lowest binding energy (−8.82 kcal mol^−1^). These structural modifications not only improved binding affinity and stability but also enhanced predicted ADME and safety profiles, suggesting their potential as next-generation TS inhibitors (Fig. 5G). Compound 21 interacts primarily with Tyr258 and His195, whereas compound 22 shows interactions with Cys195, Arg215, Tyr258, His258, and Ser216 (SI, Fig. S2F).

A related class of curcumin-5-FU hybrid pyrimidinediones was synthesized to merge thioredoxin reductase (TrxR) inhibition from curcumin with TS inhibition from 5-FU.^121^ The resulting compound 23 displayed strong selectivity toward A549 cancer cells and minimal toxicity against THLE normal cells. The inclusion of Michael acceptor groups from the curcumin moiety increased TrxR binding and ROS generation, promoting apoptosis (Fig. 5H). The combined targeting of TrxR and TS produced a synergistic anticancer effect with reduced systemic toxicity. *In vivo* studies confirmed significant tumor volume and weight reduction, validating this compound as a promising dual-action hybrid for further development. Compound 23 binds to the active site of TS, interacting with Arg50, Arg176, Leu192, Cys195, Arg215, and Ser216 (SI, Fig. S2G).

Another novel set of pyrimidine–morpholine hybrids was synthesized and evaluated against SW480 and MCF-7 cell lines.^122^ Among these, compound 24 exhibited the highest activity with IC_50_ values of 5.10 µM (SW480) and 19.60 µM (MCF-7), inducing cell-cycle arrest and apoptosis. SAR investigations revealed that electron-withdrawing substituents such as CF_3_, F, Br, and CN at the *para* position of the phenyl ring enhanced activity, with compound 24 (*p*-CF_3_) being the most potent due to increased lipophilicity and stability. Conversely, electron-donating or bulky groups, such as OCH_3_ or NO_2_, reduced potency due to steric hindrance and lower polarity, such as compound 25 (Fig. 5I). Computational (DFT and ADME) analyses confirmed that compound 24 was thermodynamically stable and possessed favorable pharmacokinetic properties, supporting its candidacy as a viable anticancer agent. Compound 24 forms hydrogen bonds with key residues His196 and Asn226. In contrast, most interactions of compound 25 with the receptor are concentrated around its trifluorophenyl group, which engages Cys195, Phe225, Ile108, Met311, and Leu213 through π interactions (SI, Fig. S2H).

Also, a series of pyrazolo[3,4-*d*]pyrimidine derivatives was developed as multifunctional antifolate analogs targeting both TS and DHFR.^123^ Compound 26 was the most potent analog, displaying broad-spectrum antiproliferative activity across NSCLC, CNS, ovarian, prostate, colon, melanoma, breast, and renal cancer cell lines, with high selectivity and non-lethal cytostatic effects. Mechanistic studies revealed that compound 26 induced S-phase arrest and apoptosis, exhibiting dual enzyme inhibition with IC_50_ = 2.41 µM (DHFR) and 8.88 µM (TS). Docking analyses indicated that N1-*p*-bromophenyl and C3-methyl substituents established strong hydrophobic interactions within the enzyme pocket. SAR trends demonstrated that C3-methyl substitution significantly increased inhibitory potency, while N1-*p*-bromophenyl substitution enhanced lipophilicity and anticancer activity. Furthermore, replacement of the pteridine core with a pyrazolo[3,4-*d*]pyrimidine scaffold improved activity, and *p*-bromophenyl substitution at C4 amplified potency across multiple cell lines, emphasizing the critical role of hydrophobic aromatic substitutions in TS inhibitor design (Fig. 5J). The NH group of the acetamide bridge forms a hydrogen bond with Asp21, while the benzoyl ring engages in pi-alkyl hydrophobic interactions with Pro61 and Pro26. Additionally, the benzoylglutamate tail of 26 establishes two hydrogen bonds *via* its C-carboxylate group with Asn64 and Lys63 (SI, Fig. S2I).

A novel series of uracil–1,2,3-triazole hybrids was designed *via* a molecular assembly approach combining pharmacophores of known TS inhibitors.^124^ Among the synthesized compounds, compound 27 exhibited the highest potency with IC_50_ = 1.18 µM against A549 cells and TS inhibitory IC_50_ = 0.13 µM, surpassing pemetrexed (IC_50_ = 3.29 µM for A549 and 2.04 µM for *h*TS). Compound 27 induced G1/S phase cell-cycle arrest and apoptosis, accompanied by downregulation of cyclin D1 and cyclin E, activation of caspase-3, and a reduced Bcl-2/Bax ratio. SAR analysis indicated that chloro substitution on the uracil-triazole scaffold enhanced antiproliferative and TS inhibitory activity. The combined presence of uracil and triazole pharmacophores, along with electron-withdrawing substitution, accounted for its superior TS inhibitory capacity (Fig. 5K). Molecular docking (PDB: 1JUJ) revealed that compound 27 binds similarly to pemetrexed, forming hydrogen bonds with Arg50 and Ala312 and π–π stacking with Phe225, which compensated for the absence of the Lys77-Glu residue hydrogen bond (SI, Fig. S2J).

A series of quinoxalinone-linked pyrazole hybrids (28–31) was synthesized through a multitarget-directed design strategy and evaluated for cytotoxicity against MCF-7, HCT-116, and A549 cell lines, as well as for inhibition of TS, BRaf, and EGFR kinases.^125^ Among them, compound 31 emerged as the most potent, exhibiting IC_50_ values of 2.04 µM (MCF-7), 2.69 µM (HCT-116), and 1.93 µM (A549), along with TS, BRaf, and EGFR inhibitory IC_50_ values of 1.16, 1.28, and 1.93 µM, respectively. All compounds complied with Lipinski's rule, suggesting good oral bioavailability. SAR analysis revealed that electron-withdrawing substituents such as NO_2_, F, Cl, and Br at the 4-position of the pyrazole ring enhanced both cytotoxic and TS inhibitory activities, while electron-donating groups like Me, Et, and OH diminished potency. The nitro-substituted compound 31 was the most active derivative, displaying superior tumor selectivity with SI values of 9.72, 7.37, and 10.27 against MCF-7, HCT-116, and A549 cells, respectively, outperforming pemetrexed (Fig. 5L). Compound 31 forms hydrogen bonds with Ala197, Leu198, and Tyr213, while also engaging in hydrophobic interactions with His196, Cys199, Gln200, Gln211, Leu212, Gln214, Arg215, His250, Thr251, Leu252, and Gly253 (SI, Fig. S2K). Overall, quinoxalinone-pyrazole hybrids, particularly compound 31, represent a promising multitarget scaffold with potent TS inhibition and enhanced selectivity over clinical standards. Table 3 illustrates a summary of pyrimidine-based thymidylate synthase inhibitors.

**Table 3:** Summary of pyrimidine-based thymidylate synthase inhibitors

Compound(s)	Scaffold/type	IC_50_ (TS or cytotoxicity)	SAR highlights	References
11	6-Aryl-5-cyano-pyrimidines	3.89–15.74 nM	Hydrazide at position 2 enhanced H-bonding and apoptosis	114
12	2,4-Deoxy-1,2,3,4-tetrahydropyrimidine-5-sulfonamides	Superior to PTX (17.21 nm)	m-CH_3_ or F enhanced potency; Cl/Br decreased activity	115
13, 14	Pyrido[2,3-*d*]pyrimidines	20.47–13.48 nM (TS)	Electron-donating groups ↑ potency; *para* > *meta* > *ortho*	116
15	Cyanothiouracil-based 2-thiopyrimidines	10.11 µM (MCF-7)	*Para*-F substitution improved potency/selectivity	117
16–19	Pyrimido[4,5-*b*]indole-2,4-diamines	Nanomolar range	2′-Naphthyl or 2′,5′-dimethoxy groups enhanced π–π stacking	118
20	2-Amino-4-oxo-6-substituted pyrrolo[2,3-*d*]pyrimidines	Submicromolar (with greater than 80% inhibition of colony formation at the highest concentration)	3-Carbon linker optimal; multitarget inhibition of TS, GARFTase, AICARFTase	119
21, 22	Pyrimidinedione-thiourea-benzoyl hybrids	−8.82 kcal mol^−1^ binding (*in silico*)	Thiourea/benzoyl improved H-bonding, π–π stacking, and ADME	120
23	Curcumin-5-FU hybrid pyrimidinedione	Strong *vs.* A549	Dual TrxR/TS inhibition; low toxicity to normal cells	121
24, 25	Pyrimidine–morpholine hybrids	5.10 µM (SW480)	*p*-CF_3_ substitution ↑ potency; EWG > EDG groups	122
26	Pyrazolo[3,4-*d*]pyrimidine derivatives	8.88 µM (TS)	*N*1-*p*-BrPh + C3-Me ↑ potency; hydrophobic aromatic groups critical	123
27	Uracil–1,2,3-triazole hybrids	0.13 µM (TS)	Cl substitution ↑ activity; strong H-bonding with Arg50, Ala312	124
28–31	Quinoxalinone–pyrazole hybrids	1.16 µM (TS)	*p*-NO_2_ > F, Cl, Br; multitarget TS/BRaf/EGFR inhibition	125

## 3 Future directions and emerging opportunities

While TS inhibitors remain cornerstone agents in cancer chemotherapy, persistent challenges—including resistance, off-target toxicity, and limited tumor selectivity—continue to limit their therapeutic impact.^126,127^ Recent developments in medicinal chemistry, structural biology, and cancer pharmacology highlight several promising directions that may reshape TS-targeted therapy.^128,129^

### 3.1 Next-generation prodrugs and modified fluoropyrimidines

Classic fluoropyrimidines such as 5-FU require multi-step metabolic activation and are rapidly degraded by dihydropyrimidine dehydrogenase (DPD), resulting in variable efficacy and dose-limiting toxicities.^130,131^ Next-generation prodrugs seek to overcome these constraints by improving metabolic stability, tumor selectivity, and pharmacokinetic properties.^132,133^

**Fig. 6:**
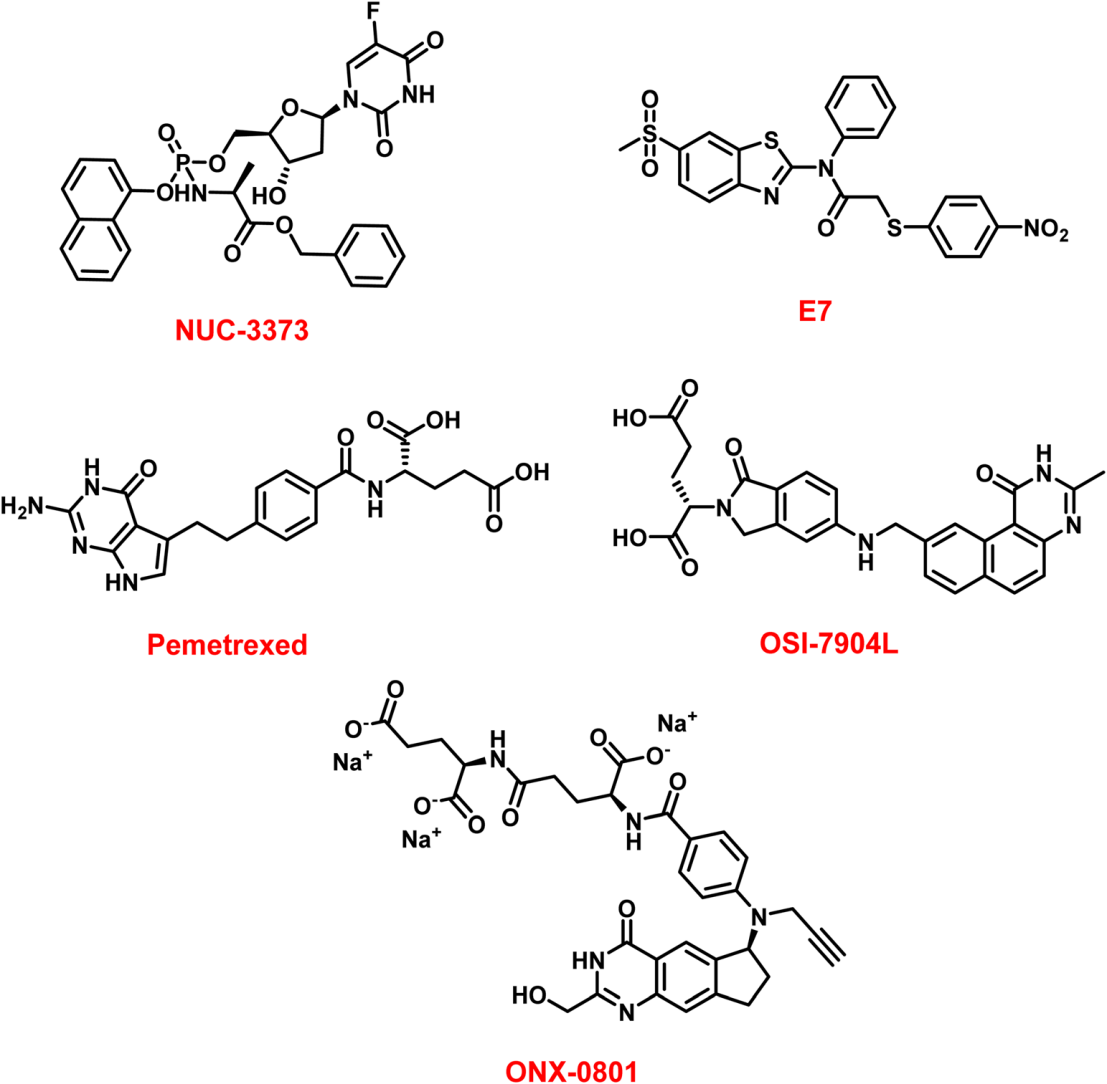
Emerging thymidylate synthase-inhibiting agents.

A leading example is NUC-3373 (Fig. 6), a phosphoramidate derivative of fluorodeoxyuridine designed to bypass DPD-mediated catabolism.^134^ Preclinical studies show that NUC-3373 produces significantly higher intracellular FdUMP levels, stronger TS inhibition, and reduced RNA misincorporation compared with 5-FU.^65,128^ In phase I evaluation, NUC-3373 demonstrated favorable pharmacokinetics, improved tolerability, and evidence of disease stabilization in heavily pretreated patients.^128^ Its DPD-independent activation and extended half-life allow shorter infusion schedules and may reduce the frequency of fluoropyrimidine-related adverse effects.^128^ Future prodrugs may incorporate tumor-specific activation triggers, targeted delivery systems, or enhanced stability to further refine clinical utility.^135^

An additional approach involves liposomal formulations of TS inhibitors, exemplified by OSI-7904L (Fig. 6), which improves drug exposure and pharmacokinetics relative to free OSI-7904.^136,137^ Similarly, folate-receptor-targeted antifolates such as ONX-0801 (Fig. 6) exploit FRα overexpression in certain tumors to achieve selective uptake and reduced systemic toxicity.^138,139^

#### 3.1.1 Challenges and future directions

(a) Designing prodrugs that integrate tumor-specific enzymatic, reductive, or pH-sensitive activation mechanisms.

(b) Leveraging nanocarriers, liposomes, or antibody–drug conjugates to improve tumor delivery.

(c) Enhancing metabolic stability while preventing accumulation of toxic intermediates.

### 3.2 Destabilizers and noncatalytic targeting

A major emerging paradigm involves targeting noncatalytic regions of TS, particularly the dimer interface, to destabilize the active homodimer rather than inhibiting the catalytic site.^5^ Recent studies describe small molecules that bind to the TS dimer interface, shifting the equilibrium toward inactive monomers, promoting proteasomal degradation, and decreasing intracellular TS abundance.^140^ Compound E7 (Fig. 6) exemplifies this “dimer destabilizer” mechanism and offers a strategy to overcome resistance driven by TS overexpression during conventional inhibitor therapy.^135,141^

This concept is supported by complementary structural efforts demonstrating that interface-binding ligands can suppress TS levels and inhibit tumor growth independently of classical catalytic inhibition.^142^

#### 3.2.1 Challenges and future directions

(a) Ensuring selective TS destabilization without perturbing unrelated protein–protein interfaces.

(b) Demonstrating robust *in vivo* antitumor efficacy and pharmacokinetic suitability.

(c) Combining destabilizers with catalytic inhibitors or hybrid molecules to produce synergistic TS suppression.

### 3.3 Multi-targeting and hybrid molecules

Tumors frequently bypass TS inhibition through metabolic rewiring, compensatory signaling, or activation of survival pathways.^143^ Hybrid molecules designed to simultaneously inhibit TS and complementary oncogenic targets are emerging as a strategy to enhance potency and limit resistance.^144^

Medicinal chemistry studies have developed bifunctional TS-EGFR inhibitors, which reduce proliferation, migration, and angiogenesis in multiple cancer models.^145^ Similarly, multifunctional antifolates such as pemetrexed inhibit TS, DHFR, and GARFT, limiting the capacity for metabolic bypass and demonstrating durable clinical benefit.^14^

Beyond classical scaffolds, recent efforts have produced novel TS-inhibitory chemotypes, including 2-thiopyrimidine-5-carbonitrile derivatives, which reduce TS expression and suppress tumor cell proliferation, migration, and 3D spheroid formation in preclinical studies.^117,146,147^ Some representatives additionally induce mitochondrial apoptosis and anti-angiogenic activity, highlighting the potential of hybrid or multifunctional designs.

#### 3.3.1 Challenges and future directions

(a) Balancing potency across multiple targets without compounding systemic toxicity.

(b) Using SAR-guided design to optimize hybrid structures for selectivity, permeability, and metabolic stability.

(c) Exploring combination regimens with immunotherapies and targeted agents.

### 3.4 Biomarker-guided and precision therapy

As therapeutic options expand, precision oncology approaches are increasingly important for optimizing TS-targeted regimens. Biomarkers such as TYMS expression, DPYD genotype, and plasma levels of TS or DHFR mRNA may help predict response or toxicity, guiding dose selection and patient stratification.^148,149^

For example, in NSCLC, TS expression may influence sensitivity to pemetrexed (Fig. 6), though its predictive value remains under investigation.^150,151^ Circulating mRNA biomarkers have shown potential to predict outcomes and may facilitate real-time monitoring of therapeutic response.^152,153^

#### 3.4.1 Challenges and future directions

(a) Standardizing TS-related biomarker assays for clinical decision-making.

(b) Integrating multi-omic biomarkers to better define responsive patient subsets.

(c) Incorporating biomarker-guided strategies into trials of emerging TS inhibitors and hybrid designs.

Fig. 6 and Table 4 summarize emerging TS-targeted therapies and clinical/preclinical status.

**Table 4:** Emerging TS-targeted therapies and clinical/preclinical status

Compound/strategy	Mechanism	Target(s)	Clinical status	Cancer type(s)	Key feature/advantage	References
NUC-3373	Prodrug; TS inhibition	TS	Phase I	Solid tumors	DPD-resistant activation; high FdUMP accumulation	134
Compound E7	TS dimer destabilizer	TS dimer interface	Preclinical	Various	Promotes TS protein degradation	135 and 141
Hybrid TS-EGFR molecules	Multi-target inhibitor	TS + EGFR	Preclinical	Lung, colon	Reduces proliferation and angiogenesis	145
Pemetrexed	Multifunctional antifolate	TS, DHFR, GARFT	Approved	NSCLC, mesothelioma	Multi-enzyme targeting, clinically validated	150 and 151
OSI-7904L	Liposomal TS inhibitor	TS	Early clinical	Solid tumors	Improved PK profile and exposure	136 and 137
ONX-0801	Folate-receptor–targeted TS inhibitor	TS (FRα-selective uptake)	Phase I	Ovarian, endometrial	Tumor-selective delivery *via* FRα	138 and 139
2-Thiopyrimidine-5-carbonitrile derivatives	Novel TS-inhibitory chemotype	TS	Preclinical	Breast, lung, liver	Multi-mechanistic effects; reduced TS expression	117, 146 and 147

## 4. Conclusion

Thymidylate synthase (TS) continues to represent a pivotal target in anticancer drug discovery, bridging fundamental enzymology with contemporary medicinal chemistry. Classical TS inhibitors, including fluoropyrimidines and antifolates, have long validated the therapeutic value of disrupting folate-dependent one-carbon metabolism. Nevertheless, the persistent challenges of resistance, dose-limiting toxicity, and limited tumor selectivity underscore the need for innovative molecular design. Recent progress in heterocyclic and hybrid scaffold development—particularly those incorporating oxadiazole, pyrimidine, and related frameworks—has yielded compounds with improved potency, selectivity, and pharmacokinetic behavior. Structure–activity relationship (SAR) studies have revealed that fine-tuning electronic distribution, substituent topology, and conformational flexibility can significantly modulate enzyme affinity and cellular response. Moreover, emerging concepts such as enzyme destabilization, multitarget inhibition, and biomarker-driven precision therapy are expanding the scope of TS-directed intervention beyond conventional active-site blockade. Collectively, these advances reflect a transition from empirical approaches toward rational, structure-guided optimization. Continued integration of computational modeling, structural biology, and translational pharmacology is expected to accelerate the development of next-generation TS inhibitors capable of overcoming resistance, reducing systemic toxicity, and achieving durable clinical benefit.

## Author contributions

Conceptualization and supervision: Ahmed A. Al-Karmalawy; data collection, data curation, visualization, methodology, and writing–review & editing: Ahmed A. Al-Karmalawy, Mohamed E. Eissa, Tarek A. Yousef, Arwa Omar Al Khatib, Samia S. Hawas. All authors approved the submitted version of the manuscript.

## Conflicts of interest

The authors declared no conflict of interest.

## Supplementary Material

RA-016-D5RA08381H-s001

## Data Availability

No primary research results, software, or code have been included, and no new data were generated or analysed as part of this review. Supplementary information (SI) is available. See DOI: https://doi.org/10.1039/d5ra08381h.
