# Supplementary material for: Bridging mechanism and design: modern medicinal chemistry approaches to thymidylate synthase inhibitors
Source: RSC Adv. 2026 Jan 16;16(4):3564–79. doi: 10.1039/d5ra08381h (PMC12809673; doi:10.1039/d5ra08381h)
Supplement: RA-016-D5RA08381H-s001 [file RA-016-D5RA08381H-s001.pdf]

Supplementary Information (SI) for RSC Advances.
This journal is © The Royal Society of Chemistry 2025

Bridging Mechanism and Design: Modern Medicinal Chemistry Approaches to Thymidylate Synthase Inhibitors

Ahmed A. Al-Karmalawy[1,*], Mohamed E. Eissa[2], Tarek A. Yousef[2,*], Arwa Omar Al Khatib[3], Samia S. Hawas[4]

[1] Department of Pharmaceutical Chemistry, College of Pharmacy, The University of Mashreq, Baghdad 10023, Iraq.

[2] College of Science, Chemistry Department, Imam Mohammad Ibn Saud Islamic University (IMSIU), Riyadh 11623, Saudi Arabia.

[3] Faculty of Pharmacy, Hourani Center for Applied Scientific Research, Al-Ahliyya Amman University, Amman, Jordan.

[4] Department of Pharmaceutical Chemistry, Faculty of Pharmacy, Horus University-Egypt, New Damietta 34518, Egypt.

* Correspondence:

Ahmed A. Al-Karmalawy: Email: akarmalawy@horus.edu.eg

Tarek A. Yousef: Email: tayousef@imamu.edu.sa

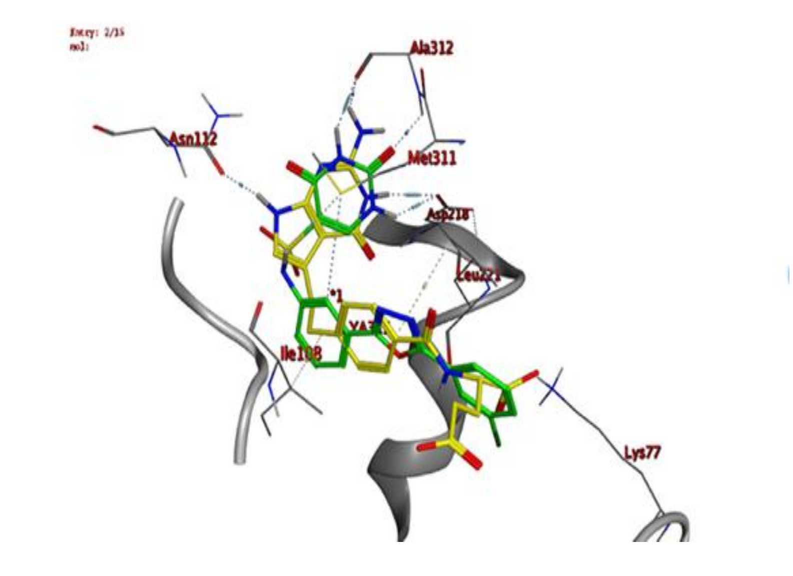	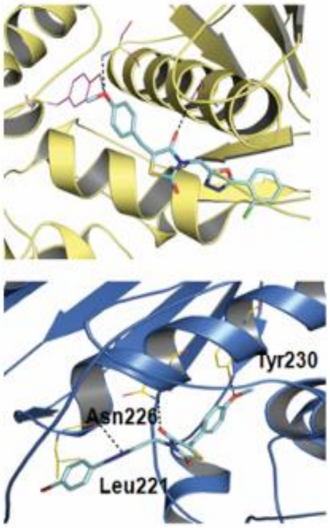
**A. Docking pose of PTX and compound 1 in TS (PDB ID: 1JUJ):**<br>PTX (green) and compound **1** (yellow) bound within the TS active site [1].	**B. Docking of compounds 2 (top) and 3 (bottom) with TS (PDB ID: 6QXG) [2].**

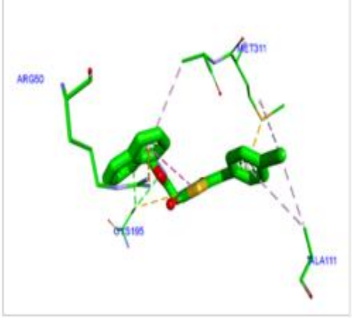	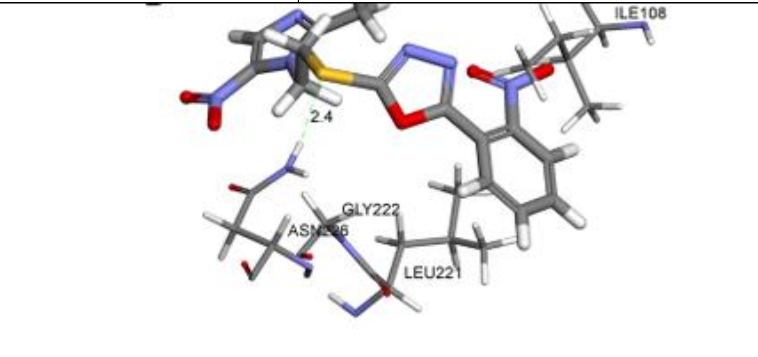
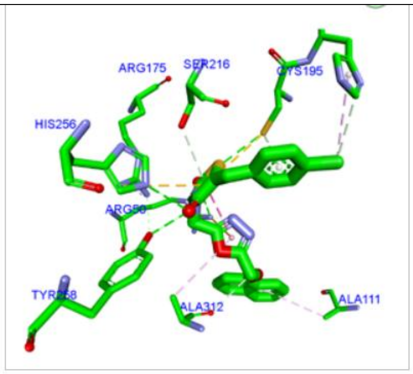	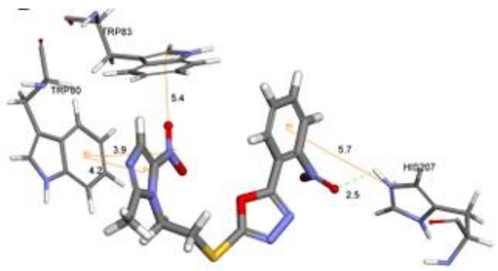
**C. Docking of compounds 4 (top) and 5 (bottom) with TS (PDB ID: 6QXG) [3].**	**D. Interaction profiles of compound 6 in TS:**<br>Human TS (PDB ID: 1HVY, top) and *E. coli* TS (PDB ID: 2KCE, bottom) showing the binding interactions of compound 6 with each target [4].

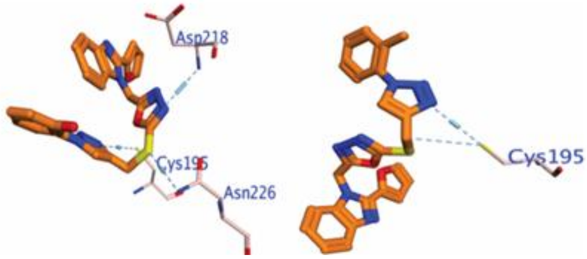	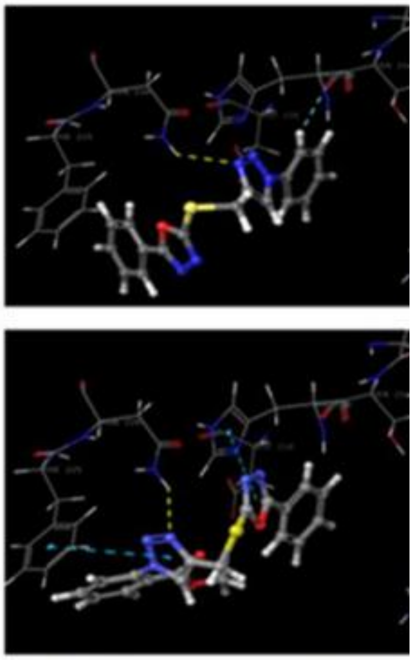
**E. Binding interactions of compounds 7 (left) and 8 (right) with TS (PDB ID: 6QXG) [5].**	**F. Docking of compounds 9 (top) and 10 (bottom) with TS (PDB ID: 6QXG) [6].**

Figure S1. Docking of compounds **1-10** with the thymidylate synthase protein.

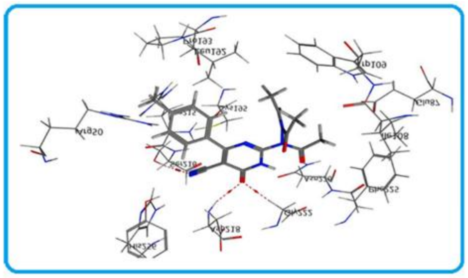	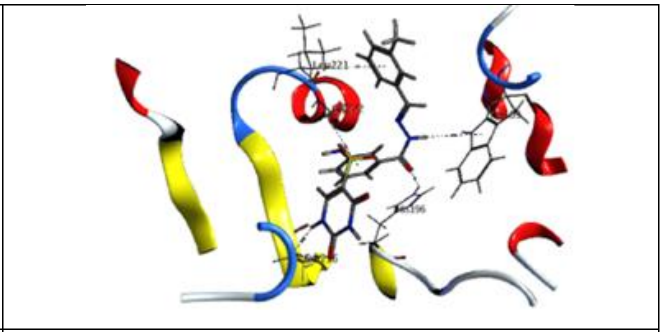
**A. Predicted binding mode of compound 11 within the TS active site (PDB ID: 6QXG) [7].**	**B. Docking pose of compound 12 in the TS co-complex (PDB ID: 1JUJ) [8].**
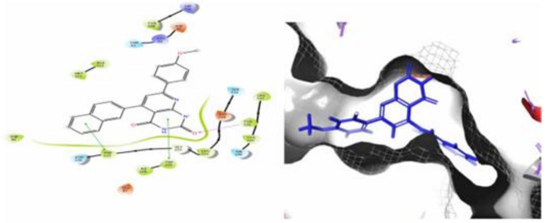<br>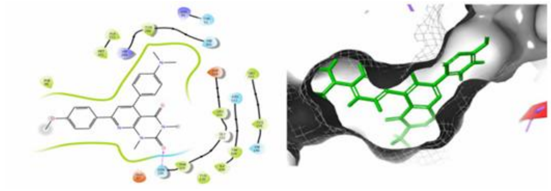	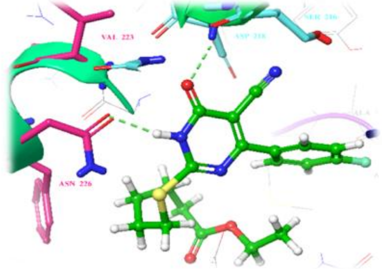
**C. Docking orientations of compounds 13 (top) and 14 (bottom) in human TS (PDB ID: 1HVY) [9].**	**D. Docking of compound 15 with TS (PDB ID: 1JU6) [10].**
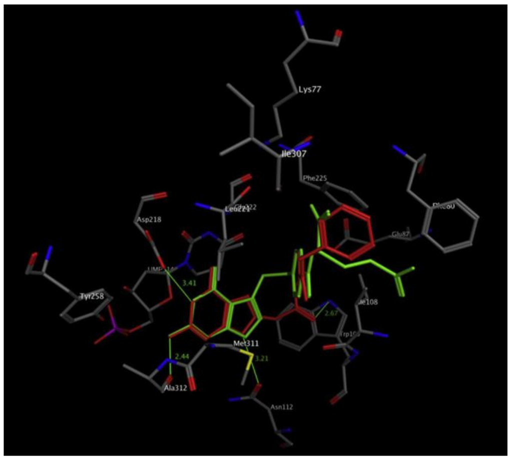	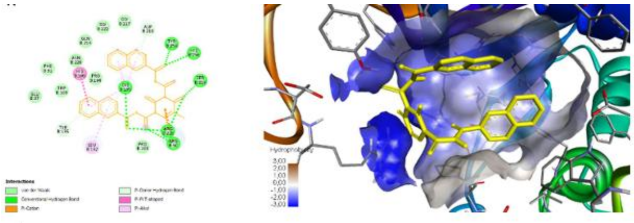<br>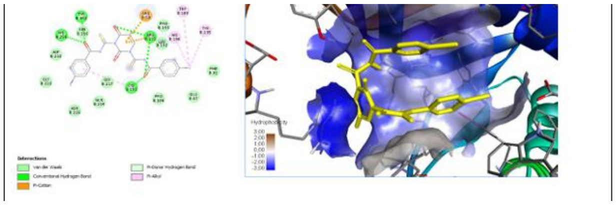

<table>
<tr><td>E. Overlay of compound 20 (red) and PMX (green) in human TS (PDB ID: 1JU6) [11].</td><td>F. Docking of compounds 21 (top) and 22 (bottom) with TS (PDB ID: 6QXG) [12].</td></tr>
<tr><td>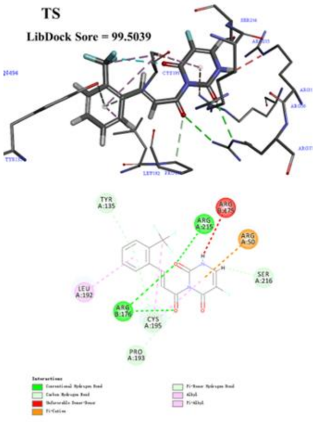</td><td>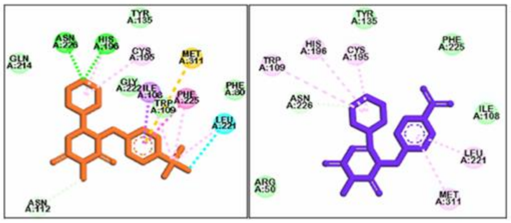</td></tr>
<tr><td>G. Docking of compound 23 with TS (PDB ID: 6QXG) [13].</td><td>H. Docking of compounds 24 (left) and 25 (right) with human TS (PDB ID: 1HVY) [14].</td></tr>
<tr><td>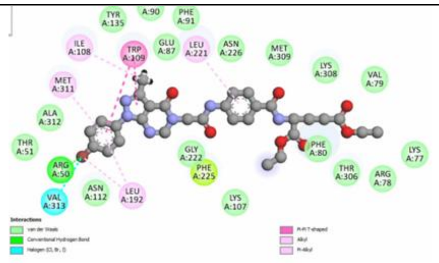</td><td>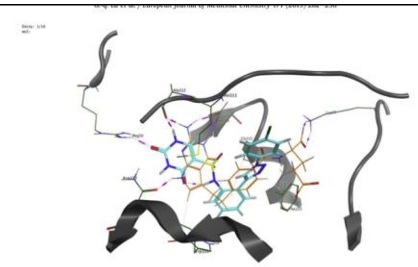</td></tr>
<tr><td>I. Docking of compound 26 in the TS active site (PDB ID: 1JU6) [15].</td><td>J. Docking of compound 27 (blue) and PTX (orange) in the TS binding pocket (PDB ID: 1JUJ) [16].</td></tr>
<tr><td colspan="2">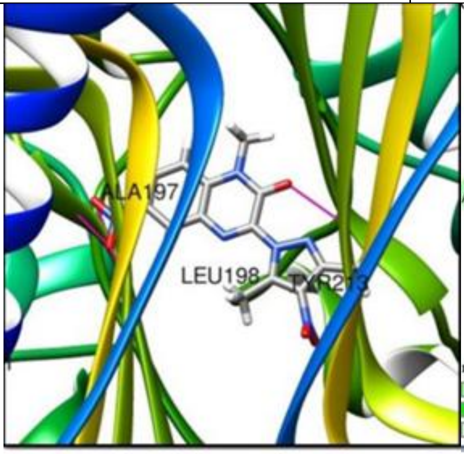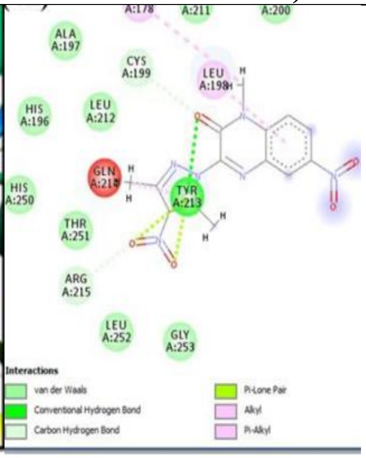</td></tr>
<tr><td colspan="2">K. Docking of compound 31 with TS (PDB ID: 1JUJ) [17].</td></tr>
</table>

Figure S2. Docking of compounds **11-31** with the thymidylate synthase protein.

References

1. X.-y. Li, D.-p. Wang, G.-q. Lu, K.-l. Liu, T.-j. Zhang, S. Li, K. Mohamed O, W.-h. Xue, X.-h. Qian and F.-h. Meng, *Journal of advanced research*, 2020, **26**, 95-110.
2. Z. M. M. Alzhrani, M. M. Alam, T. Neamatallah and S. Nazreen, *Journal of Enzyme Inhibition and Medicinal Chemistry*, 2020, **35**, 1116-1123.
3. S. Nazreen, *Archiv der Pharmazie*, 2021, **354**, e2100021.
4. Q.-R. Du, D.-D. Li, Y.-Z. Pi, J.-R. Li, J. Sun, F. Fang, W.-Q. Zhong, H.-B. Gong and H.-L. Zhu, *Bioorganic & Medicinal Chemistry*, 2013, **21**, 2286-2297.
5. A. S. Almalki, S. Nazreen, S. E. I. Elbehairi, M. Asad, A. A. Shati, M. Y. Alfaifi, A. Alhadhrami, A. A. Elhenawy, A. Q. Alorabi and A. Asiri, *New Journal of Chemistry*, 2022, **46**, 14967-14978.
6. M. M. Alam, A. S. Almalki, T. Neamatallah, N. M. Ali, A. M. Malebari and S. Nazreen, *Journal*, 2020, **13**.
7. L. H. T. Amin, T. Z. Shawer, A. M. El-Naggar and H. M. A. El-Sehrawi, *Bioorganic chemistry*, 2019, **91**, 103159.
8. G. Dong, Y.-h. Li, J.-s. Guo, Q.-q. Lin, M.-y. Deng, W.-h. Xue, X.-y. Li and F.-h. Meng, *European Journal of Medicinal Chemistry*, 2023, **258**, 115600.
9. A. Kumar, N. Backer, H. Paliwal, A. K. Singh, T. Debbaraman, V. Singh and P. Kumar, *BMC Chemistry*, 2024, **18**, 161.
10. A. Jaitak, K. Jangid, R. Singh and V. Monga, *European Journal of Medicinal Chemistry*, 2026, **301**, 118205.
11. Y. Liu, C. Zhang, H. Zhang, M. Li, J. Yuan, Y. Zhang, J. Zhou, H. Guo, L. Zhao, Y. Du, L. Wang and L. Ren, *European Journal of Medicinal Chemistry*, 2015, **93**, 142-155.
12. R. Ruswanto, I. I. Tita Nofianti, R. Mardianingrum, S. Fajriah, A. R. Aulia, Z. P. Handirana and S. F. Zahwa, *Journal of Pharmacy Pharmacognosy Research*, 2025, **13**, S83-S99.
13. X. Wang, X. Li, X. Zhang, X. Wang, J. Yang and G. Liu, *Biochemical Pharmacology*, 2024, **230**, 116559.
14. E. Ataollahi, L. Emami, A. M. Al-Dies, F. Zare, A. Poustforoosh, M. Emami, F. Saadat, F. Motamen, Z. Rezaei and S. Khabnadideh, *Frontiers in chemistry*, 2025, **13**, 1537261.
15. M. Mahnashi, M. M. Alshahrani, A. Al Ali, A. Asiri and M. A. Abou-Salim, *Journal of Enzyme Inhibition and Medicinal Chemistry*, 2023, **38**, 2203879.
16. G.-q. Lu, X.-y. Li, K. Mohamed O, D. Wang and F.-h. Meng, *European Journal of Medicinal Chemistry*, 2019, **171**, 282-296.
17. C. E. Theodore, A. M. Anusuya, G. Sivaiah, R. Jain, C. S. A. Kumar, S. B. B. Prasad, M. S. Raghu, F. A. Alharti, M. K. Prashanth and B.-H. Jeon, *Journal of Molecular Structure*, 2023, **1288**, 135765.
